# Computations in living organisms modeled by marked graphs

**DOI:** 10.1007/s11538-025-01499-x

**Published:** 2025-07-25

**Authors:** John M. Myers, Hadi Madjid

**Affiliations:** 1 John A. Paulson School of Engineering and Applied Sciences (Retired), Harvard University, 29 Oxford St., Cambridge, Massachusetts 02108 USA; 2 Consultant, 309 Winthrop Terrace, Bedford, Massachusetts 01730 USA

**Keywords:** Marked graphs, Live and safe markings, Slime mold, Unpredictable changes.

## Abstract

The accurate copying of nucleotides in DNA replication is arguably a digital computation. So are some cognitive capacities found in all organisms. In 2005 we proved that linking quantum calculations to evidence requires guesswork subject to revision (Madjid and Myers 2005). Based on this proof, we assume computations by living organisms undergo incessant unpredictable changes in their structure. This raises a question: how can changes in computations be made while preserving the integrity of the organism? We offer an answer expressed in the mathematics of marked graphs. Computations as networks of logical operations can be represented by marked graphs with live and safe markings. We represent a sequence of changes by a sequence of marked graphs. Then “Preserving the integrity of the organism” is expressed by preserving liveness and safety throughout the sequence of marked graphs. For example, we show how a single slime-mold amoeba inserts itself into a slime-mold filament without interrupting computation spread along the filament. Because interpretations of mathematics are mathematically undetermined, a quite different interpretation of the same sequence of marked graphs is possible. An alternative interpretation of the sequence of marked graphs is to see them as a cartoon of the insertion of a fragment of thought into a chain of human thoughts.

## Introduction

Cognitive processes in people and other organisms involve computations, some of which are digital. Also involving digital computation are various biochemical processes, such as DNA replication and metabolic cycles. We assume that all these processes involve, in addition to computation, something beyond computation. The basis of this assumption is a little known feature of quantum theory: Unlike Newtonian mechanics, quantum theory has the capacity to express its own relation to experiment. The language of quantum theory allows one to inquire into the relation between (1) models stated theoretical terms and (2) probabilities of experimental outcomes. In 2005 our inquiry resulted in the proof of what amounts to the necessity of guesses that reach beyond logic (Madjid and Myers 2005). That finding points to an avenue of investigation of the physicist as a biological creature that makes guesses, likely influenced by emotions and feelings. In light of biological evolution, our proof points more broadly to calculations throughout all of life as networks of logical operations interspersed with unpredictable changes in these networks. Better understanding of such networks of logical operations, subject to unpredictable changes, would be of interest, e.g. regarding cognition at various levels and also regarding the functioning of biochemical processes.

Types of logical operations include arithmetic types such as addition and multiplication. A computation typically requires more than one logical operation. Each operation consumes input(s) and produces output(s). Because outputs from some operations are transmitted as inputs to other operations, communications are intrinsic to computation. Conversely, digital communication involves a network of nodes that perform mathematical operations to process messages. Thus, **digital computation and communication are inseparable**, and we express both by networks of logical operations.

Historically, logic emphasizes relations among truth values as if these were static; however, computations depend of interconnected, dependably repetitive *motions* whether the motion of fingers pushing abacus beads or electronic motions in digital computers. By logical *operations* we mean operations that move to consume inputs and produce outputs. The outputs produced at one moment are related to the inputs at a previous moment by logical *relations*. From this point of view a logical relation is a relation between a snapshot of input and a snapshot of a later output. One can think of snapshots before and after moves of a play of chess.

We wondered about computation in a developing brain, in which cells move past one another in an organized way. Could we express something of such motion in terms of networks of logical operations and their unpredictable changes? That wonder led us the simpler example of free-swimming amoebae that come together to form the filaments of a slime mold (Bonner 2009), discussed below. We want to represent: (1) the logical structure of computations in living organisms, and (2) the unpredictable changes in these computations. To this end, in section 2 we tailor marked graphs with live and safe markings. The graphs are comprised of nodes representing logical operations, along with arrows that connect some nodes to other nodes. Graphs act as game boards on which some of the arrows are marked by tokens; the tokens are moved to represent the execution of the logical operations of a network.

A change in a network of logical operations, whether predictable or unpredictable, can be expressed by a pair of marked graphs, one before the change, the other after the change. We display a sequence of marked graphs to cartoon the way a slime-mold amoeba can insert itself into a chain of other amoebae that forms a filament of a slime mold. The point is to show how this insertion can proceed while preserving an active communications network along the filament as the amoeba is being inserted.

While the marked graphs show networks of interconnected logical operations, they give no indication of timing. This restriction is also a strength: it allows both for concurrent operations and for comparisons of computations by widely differing organisms. The restriction to logic, omitting physical implementation, also allows a given logical structure to serve widely differing purposes.

The marked graphs of section 2 show connections among operations but do not describe the operations themselves. In section 3 we remedy this deficiency by introducing numerical labels on tokens, in order to exhibit the logical operations of the marked graphs. Examples are discussed. Section 4 is a concluding discussion. Appendices supply technical definitions, remarks, and some proofs.

## Marked graphs to represent networks of mathematical operations

Several considerations lead us to choose marked graphs to represent computations in living organisms.

1. Although a clock is helpful to scientist describing motion, it is misleading to assume that any living organism has ‘clock time’ as a resource for its own functioning; hence we want a representation that avoids assuming (explicitly or tacitly) that sources of local timing conform to a system-wide clock.
2. We want a representation of computation capable of expressing any digital computation that can be executed with finite resources.
3. We want to draw on the hardware used in digital computers, which has only simple components, without depending on software concepts which are unlikely to be instantiated in most living organisms.

These desiderata lead to marked graphs—a specialization of Petri nets (Petri 1996). Marked graphs can express operations that occur in no particular temporal order, consistent with the functioning of local rhythms in organisms which have no system-wide clock.

Marked graphs consist of graphs marked by tokens. As described more formally in Appendix  A, a graph consists of nodes representing operations, along with arrows connecting certain pairs of nodes. With respect to an arrow connecting a pair of nodes, the node touched by the tail of the arrow is called the *source* of the arrow, and node at the head of the arrow is called the *target* of the arrow. We say an arrow connects its source to its target. In some cases, the source and target are the same, in which case one calls the arrow a *loop*. An arrow pointing into its target node is an *in-arrow* of that node; an arrow pointing out of its source node is an *out-arrow* of that node. An arrow is an out-arrow of its source and an in-arrow of its target. A loop is both an out-arrow and an in-arrow of the node that it connects to itself. An arrow indicates that the source produces something consumed by the target.

Dynamics of computation are expressed by markings and by moves that change one marking into another. A marking of a marked graph is a placement of tokens on certain arrows. We deal with two cases: tokens that are labeled e.g. by mathematical expressions and token that are unlabeled. For both cases the dynamics of computation are expressed by rule-bound changes in markings, defined by a *firing rule*. Both labeled and unlabeled tokens are moved according to the firing rule. The firing rule is this: **If tokens are on all the in-arrows of a node, the tokens are removed from those arrows and then tokens are placed on all the out-arrows of the node. One says the node fires.**

The graphs that we use in this report to show changes in networks of operations need not show token labels; however, section 3 shows how marked graphs with numerical labels on their tokens can represent the logical operations of digital computations of arbitrary (finite) complexity.

**Fig. 1:**
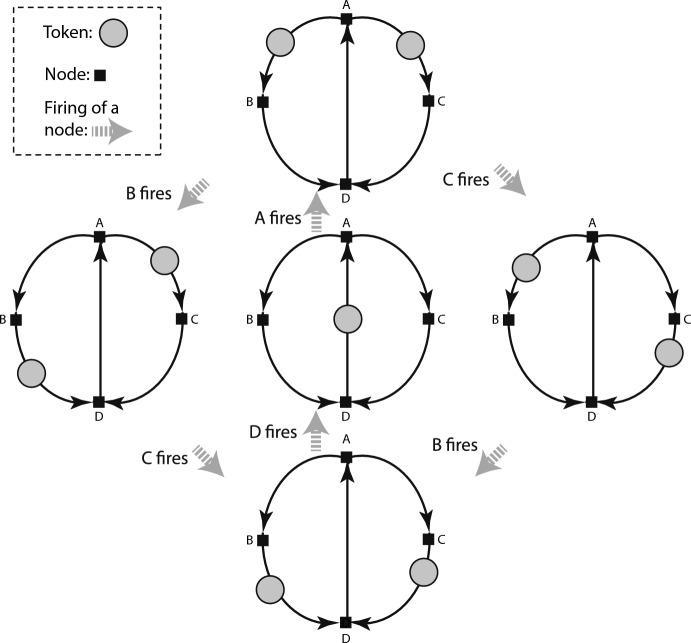
Marked graph with its markings

Figure 1 shows an example of a marked graph with unlabeled tokens.

Starting with the middle graph, node A is fireable, and firing it produces the top marked graph, in which nodes B and C are concurrently fireable. If B fires and then C fires the right hand marking is obtained and then the bottom marking; conversely if C fires and then B fires, the left hand marking is obtained and then the bottom marking. Either way, when D fires, the middle marking is repeated.

The top marking makes both nodes B and C fireable, imposing no temporal order on their firings. B and C are said to be *concurrently* fireable in that marking. The arrows of a marked graph show the logically necessary relations. The capacity to express node firings that are partially rather than totally ordered is one of several advantages of marked graphs. If, instead of marked graphs, one indicated a clock reading for each firing, it would be unclear which relations between earlier and later clock readings were logically necessary as opposed to accidental.

A graph with an initial marking defines a *token game*. The graph is the game board, and the moves are node firings. The point of the game is not to win or lose, but to enact a small computation or to imagine and analyze a large computation, as if it could be executed on a (large) graph. One thinks of each node as having a player. For small graphs, one person may play several or all of the nodes.

### Live and safe markings come in families

An important property of the graphs that express computations is this: their markings must be such that no arrow can have more than one token (the marked graph is called *safe*), and no deadlock occurs, so that whatever marking is allowed, there is some node that can fire (the marked graph is called *live*) (Commoner et al. 1971). Live and safe markings are possible only if a graph consists of one or more components, each of which is a *strong* graph, where *strong* means that the graph contains a directed path from every node to every other node.

**Fig. 2:**
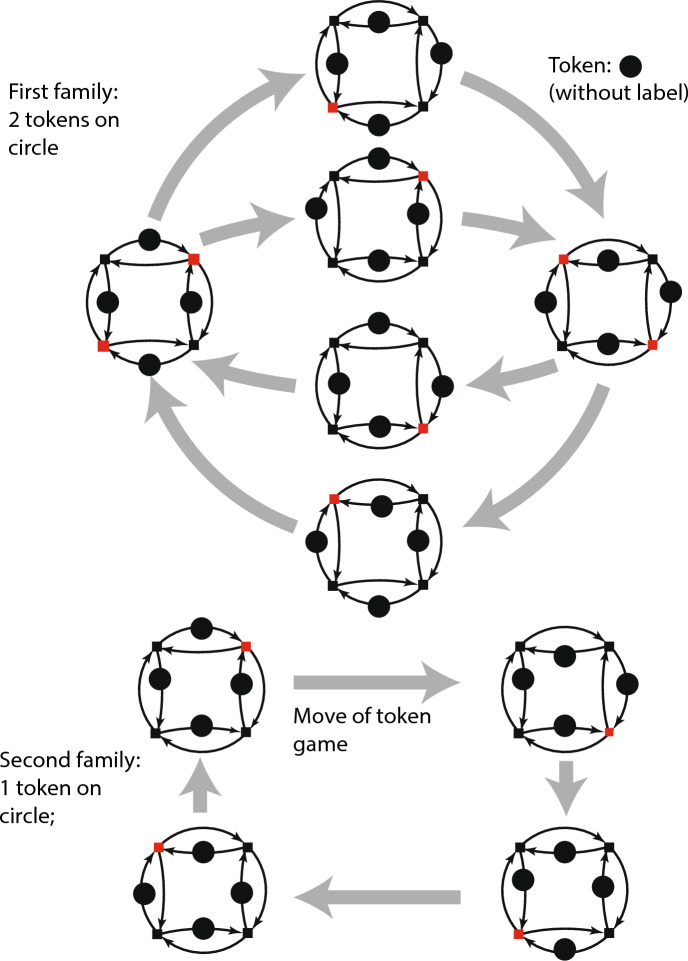
Markings of two families

Figure 2 below shows pictures of two token games played on the same graph. The two games each have live and safe markings. Nodes are small squares. Nodes that are fireable for a marking are in red. Arrows of the graph are thin and black, while moves of the token game are indicated by thick gray arrows. The ten live and safe markings shown in fig. 2 fall into two disjoint *families*, one family for each token game. The first family includes markings in which two nodes are concurrently fireable; the second family has no such markings.

The figure shows the motion of node firings—moves of the token game—in a way that reminds me of a record of a chess game that shows a snapshot of the pieces on the board before a move, paired with a snapshot showing the pieces on the board after the move. **The existence of more than one family for generic graphs implies that marked graphs with specified families convey more information that could be conveyed by unmarked graphs.**

### Marked graphs are topological

Marked graphs show what nodes have to fire to enable other nodes to fire, but they indicate nothing about the time in seconds between the firing of one node and the firing of any other node. They represent the topology, without any time metric. This restriction to network topology is a limitation and a strength. This definition specifies nothing about how quickly or slowly the production takes place. Thus, timing can be described only by means outside of networks of logical operations. (Such means are addressed in (Myers and Madjid 2024, 2020)). The omission of timing, however, is also a strength, in that it allows one to compare living computations executed by organisms that differ from each other widely and at different scales, for example comparing logical operations of a plant with those of an animal.

### Communication in a growing filament of a slime mold

**Fig. 3:**
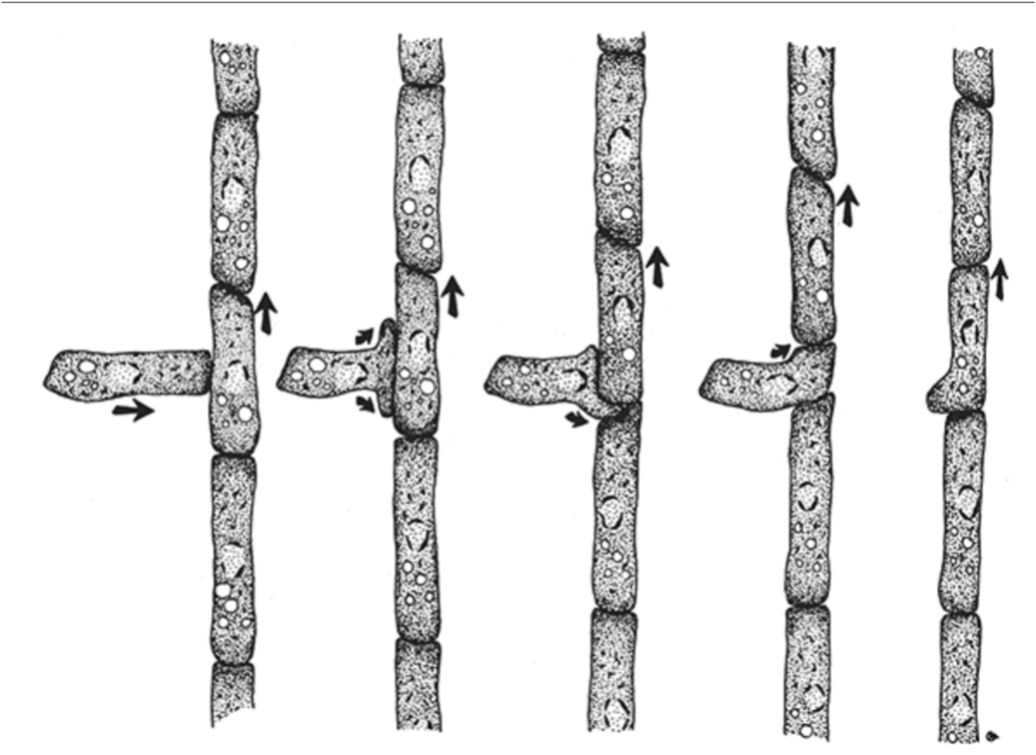
Drawn stages of entry of amoeba into a slime-mold filament, from (Bonner 2009), with permission of Princeton University Press

A sequence of drawings, reproduced in fig. 3, shows stages of the entry of an amoeba into a filament of these amoeba in the slime mold Dictyostelium discoideum. We want to express the developing filament by a sequence of marked graphs, which, like the drawing, would be “snapshots” of a continuous process taken between elementary steps, as in fig. 3. We assume that there is some kind of communication network stretched out as a chain along the filament, the insertion of an amoeba entails the insertion of a small network, that of a single amoeba, into the chain.

**Fig. 4:**
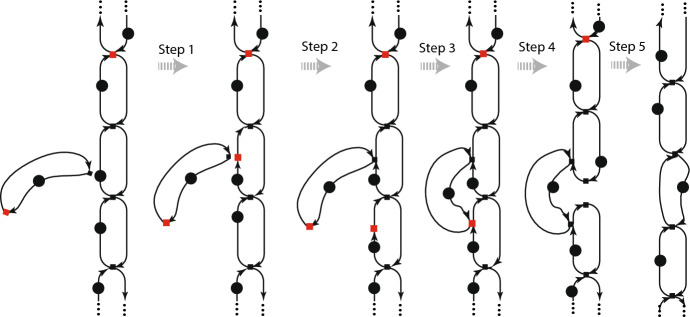
Marked graph changes representing entry of amoeba into a slime-mold filament

Each snapshot consists of a marked graph links subgraphs into a chain. Reading left to right, the beginning snapshot shows an unattached amoeba close to a chain; the ending snapshot shows the chain extended by the entry of the amoeba. We want to: (1) preserve liveness and safety over a sequence of steps: and (2) maintain an uninterrupted communication network along the filament. The following five steps achieve this:

1. Step 1 is the insertion of a node into an arrow to produce two arrows, an in-arrow and an out-arrow of the node.
2. Step 2 merges a node of the small graph for the single amoeba with a node of a link of the chain.
3. Step 3 is the merging of two nodes of a graph, a step that preserves liveness and safety if made at a marking that makes both nodes fireable.
4. Step 4 is the splitting of a node that satisfies conditions that allow the split to preserve liveness and safety.
5. Step 5 is the deletion of unneeded nodes.

Appendix  A.3 gives the proofs that each of these steps transform live and safe marked graphs to other live and safe marked graphs, so that any sequence of such steps produces a live and safe marked graph. These proofs take for granted a knowledge of basic results established by (Commoner et al. 1971). As far as we know, the proof for step 3 is original. Although fig. 4 represents the computational network of an amoeba cartooned as a mere cycle, more complex representations of an amoeba satisfy the conditions of the proofs. Indeed, the four steps work for an amoeba as represented by any live and safe

graph.

**Note** that the shapes of the marked graphs, while helping one to associate fig. 4 with fig. 3, have no mathematical content. The mathematical content of a graph with a marking is exclusively in (a) the source and target node of each arrow, and (b) the presence or absence of a token on each arrow (and also the labels on the tokens when those are included). In other words, deforming a drawing of a marked graph by bending, stretching, or shrinking arrows has no effect on the graph represented by the drawing. I.e., marked graphs express only topological relations.

### Dependence on choice of marking

**Fig. 5:**
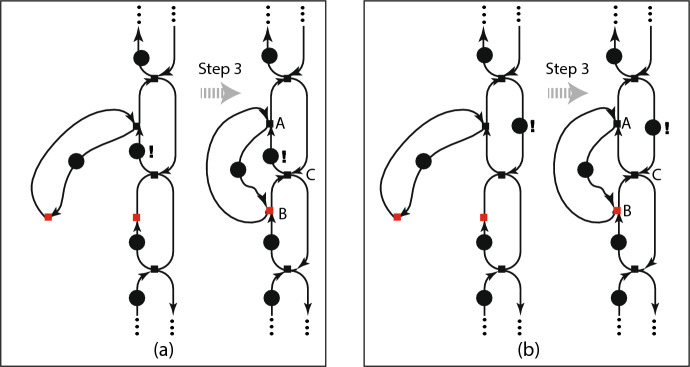
(a) Zoom of step 3; (b) Step 3 with alternate marking

Here we come to a technical point that shows how markings express something inexpressible by graphs without markings. fig. 5 zooms in on step 3 of fig. 4.

On the left (a) is the marking as in fig. 4; on the right (b) the step is shown for a different marking. The markings are the same except for the position of one token, indicated by “**!**”. That one token determines which of two cycles bears a single token and therefore imposes a cyclic order on the firing of the nodes contained in it. For (a) the nodes A, B, and C fire in the cyclic order (B,A,C), while for (B) the nodes fire in the other cyclic order (B,C,A). The imposition of a cyclic order holds until step 4.

Figure 5 has another implication. The merging of a node can be undone, so the figure shows a way for a family to change by the splitting of a node, followed by the firing of another node and merging that restores the node that was split.

## Marked graphs with labeled tokens represent the logic of computations

Marked graphs as used above show the connections between logical operations involved in a calculation but omit specifications of these operations. To express logical operation we augment the firing rule with a labeling rule for each node. For each node that represents a logical operation, the labeling rule specifies labels on tokens on out-arrows as logical functions of labels on tokens on in-arrows. Correspondingly, the token game for a marked graph with labeled tokens executes the specified labeling rules.

**Fig. 6:**
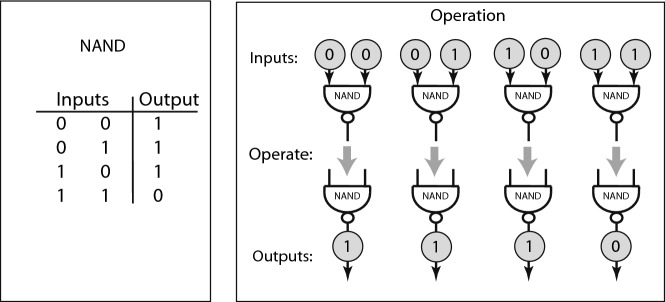
NAND

**Fig. 7:**
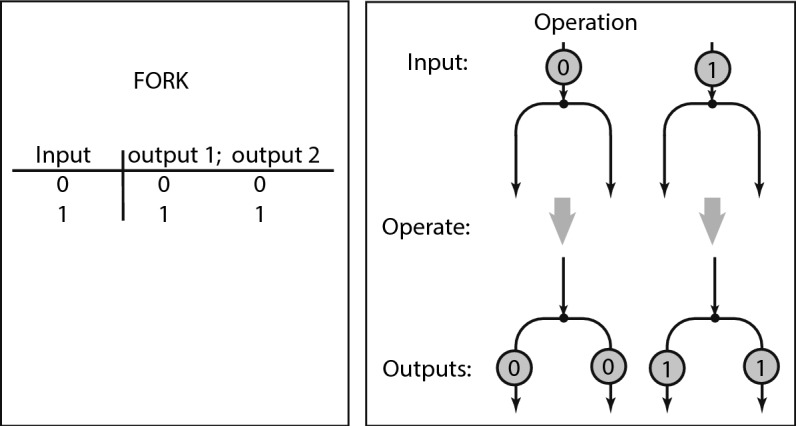
FORK

Any computation can be represented by three types of nodes, two of which have labeling rules, while the third is left without labeling rules, in order to express connections of the computation to an environment. We call the two basic nodes that have labeling rules NAND and FORK. Both of these nodes deal with token labels that are single bits: 0 or 1. Instead of the generic small black squares use to denote nodes, we denote NAND and FORK by specific symbols (borrowed from digital engineering). The logic operations specified by these nodes are shown in figs. 6 and 7.

FORK is a branching; it consumes one input and issues two outputs.

The third type of node is ENV for the environment; it is arbitrary in its labeling.

### Example of 1-bit full adder and its contraction

**Fig. 8:**
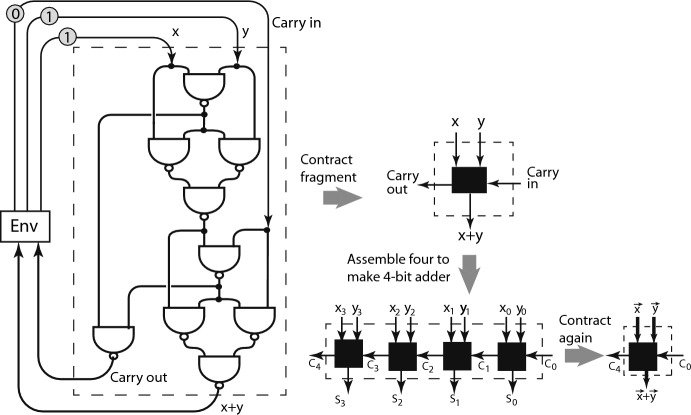
1-bit full adder, its contraction, and 4-bit adder and its contraction

To represent a laptop computer by a marked graph containing the basic types of nodes could require billions of nodes; however, morphisms come to the rescue. Appendix  B defines contractions of fragments of large graphs into single nodes smaller graphs having nodes with more complex labeling rules. For example, the lefthand part of fig. 8 shows the arrangement of NAND and FORK nodes to express the logic of the digital circuit that performs a 1-bit addition with carry. We use the convention of digital circuitry to omit some arrow heads on arrows. The omitted arrow heads all point down. The fragment shown in the dashed box on the left of the figure is drawn to its right contracted. At the bottom right of the figure four such contracted nodes are shown arranged to make a 4-bit full adder, and its contraction shown to the right, where $\vec {x}$ and $\vec {y}$ are each 4-bits.

### Relation of token games to the hardware of digital computers

Two engineering disciplines that shape digital computers are hardware and software. Of these, hardware engineers organize physical matter to execute transitions among distinct logical conditions, while the software results from efforts to make computers easy and efficient for human purposes, e.g. for programming. To represent the logic of computations made by organisms, we draw on the physical structure of computer hardware, avoiding software, because software is specialized to specifically human purposes. We model biological computations by minimal networks of computer hardware components, as if computers could be designed to specific purposes. We use marked graphs with their token games to model such minimal purpose-specific networks.

Inside a computer everything but the fan looks the same whether the computer is plugged in and running or whether it is turned off, but the hardware is in motion. This motion is not the turning of gear wheels, but the motion of electrical charges in transistors that turn each other on and off in a rapid rhythm, as if they were billions of interconnected tiny light switches. These switches manipulate transitions among distinct conditions, as do fingers moving beads on an abacus, but with electrical rather than mechanical “beads.” The distinct electrical conditions of a computer are thought of as 0 and 1. For example, computers code each letter of the alphabet with a string of 0’s and 1’s (the usual code for letter ‘a’ is the string of 8 bits: 01100001). Whatever the computer does is specified by logic functions on 0’s and 1’s, and any logic function can be instantiated by NAND and FORK operations. Hence, **any computation any computer or computer network can execute is exactly expressed by a token game played on a graph in which the nodes are of the two types NAND and FORK.** The NAND is performed by four transistors, while the FORK is performed by a branching of a wire. The formulation of marked graphs in mathematics means that properties of marked graph can be stated as propositions and proven. These properties are valid regardless of the size of the graph, and hence apply to computers, no matter how many billions of nodes they have.

### Phase detection

“...brains are foretelling devices and their predictive powers emerge from various rhythms they perpetually generate. At the same time, brain activity can be tuned to become an ideal observer of the environment, due to an organized *system* of rhythms” (Buzsáki 2006, [p. vii]).

**Fig. 9:**
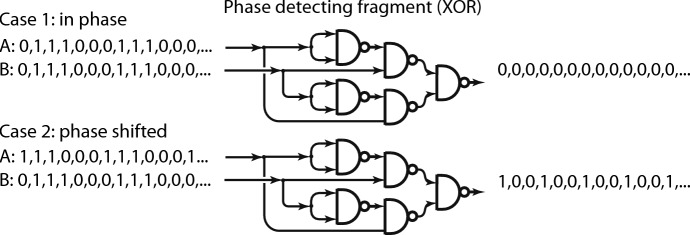
Graph fragment that detects shift in rhythms

A shift between two rhythms emanating from different clumps of brain cells can be physiologically significant. To be significant, it has to be registered. Figure 9 shows a fragment of a marked graph that responds to the shift of one rhythm relative to another. In Case 1, rhythms of 0’s and 1’ from two sources, A and B, are compared; because there is no difference between them the out-arrow receives a sequence of tokens labeled 0. In Case 2, the rhythm from source A is shift relative to that from source B, resulting in the out-arrow receiving a sequence of tokens, some of which are marked 1, indicating a difference at that position between the rhythms from A and B. The graph fragment is known in computer design as “exclusive or.”

### Logical operations in biological computations

All living organisms depend on chemical processes that manipulate the four nucleotides that make the “beads of a necklace” of DNA, so as to copy a strand of DNA into its complementary strand, with an astoundingly low error rate (Alberts et al. 2015, p. 244). What an amazing computation. Such biological computations correspond to marked graphs with labeled tokens, ultimately resolvable into NAND and FORK nodes. The marked graphs specify the logic of networks of mathematical operations that constitute computations. That specification of the logic is all that they offer. Marked graphs with unlabeled tokens specify still less. Marked graphs in this report give no indication how the mathematical operations are to be accomplished—e.g whether they are physically small or large, fast or slow, done by flesh or by silicon chips. By virtue of this restriction we can apply to biological computations insights extracted from digital computers and computer networks. We can also choose to interpret any marked graph in many different ways, re-using it analogously to the repurposing of ossicles in the evolution from fish to mammals. For example, while fig. 3 displayed the association of a sequence of marked graphs to a slime mold amoeba entering a filament, by an alternate choice one can associate the same sequence of marked graphs with the insertion of an element of human thought into chain of thoughts. This corresponds to a view of human thought as dynamically re-shapeable, a kind of dance or musical improvisation.

## Discussion

Our proof in (Madjid and Myers 2005) separates what can be calculated from what occurs but is incalculable. Here we begin to explore implications of that separation for biology. Based on the proof that calculations are accompanied by unpredictable changes, we propose that any living organism computes, punctuated by unpredictable changes in its computational structure.

Unpredictable changes in computational structures, representable by marked graphs, occur not only during the life of an organism, e.g as part of learning, but also in evolution: mutations make unpredictable changes in computational structures.

An important subtlety skated over in this paper, addressed in our (Myers and Madjid 2024), is the distinction between the specification of a logical operations, as in figs. 6 and 7, and physical machinery such as a neuron or a transistor, that moves in accordance with that specification. Incorporating the need for motion into the concept of logic would shift the concept of logic from its historical fixation on the truth values of sentences by recognizing that logic, as it is used in human and other organisms, consists in interconnected, dependably repeatable *motions*—motions essential to the organism’s functioning.

The broad conclusion of this exploration is that by recognizing calculation as accompanied by unpredictable changes, based on the proof of guesswork, one can appreciate mathematical operations as fundamental to life.

## Appendix:Mathematics of marked graphs

### Definitions

The phrase *marked graph* is used in various ways, not all of which involve token games. In our usage, marked graphs are a form of Petri net, a T-net (Petri 1996), modified to work with directed multi-graphs with loops, as explained below, and also to have tokens that carry labels of bits or strings of bits. We picture marked graphs by drawing nodes connected by arrows, with tokens on some of the arrows. For many purposes such drawings suffice, but there is a major advantage to having their mathematical definitions available, for these enable proofs of various properties of interest (Commoner et al. 1971). We define a graph mathematically to be what Harary et al. call a *net* (Harary et al. 1965, p. 5); thus a graph (also called a directed multi-graph with loops) consists of:

1. A set *V* of elements that we call “nodes.”
2. A set *X* of elements that we call “arrows.”
3. A function *s* whose domain is the set *X* of arrows and whose range is contained in *V*.
4. A function *t* whose domain is *X* and whose range is contained in *V*.

For any arrow of *X*, the function *s* assigns a node that we call the “source,” and the function *t* assigns a node that we call the “target” of the arrow. An arrow is *incident* with both its source and its target. In addition,

5. The set *V* is finite and not empty.
6. The set *X* is finite.

We say an arrow connects its source to its target. An arrow pointing into its target node is an in-arrow of that node; an arrow pointing out of its source node is an out-arrow of that node. An arrow is an out-arrow of its source and an in-arrow of its target.

Definitions pertaining to graphs vary among authors. Ours are the following:

A *directed path* in a graph is a sequence of arrows joined head to tail at nodes, with no repeated arrows.

A *simple* directed path has no repeated nodes.

A *circuit* is a closed directed path (which can be a *loop*)

A *cycle* is a closed simple directed path. (No repeated nodes.)

A graph is *strong* if it contains a directed path from every node to every other node.

An *induced subgraph* of a graph *G* is any graph consisting of a subset of nodes of *G* and all the arrows of *G* that connect pairs of those nodes.

#### [Style1 Style2]Definition 1

An *unlabeled marking* of a graph is an assignment of a (non-negative) whole number, called the *token number*, to each arrow. In this report, that number is either 0 or 1. A *labeled marking* accompanies an assignment of 1 (representing token presence) with a label consisting of a string of 0’s and 1’s (sometimes just a single 0 or 1).

A node is *fireable* in a marking if the token number on each in-arrow of the node is at least 1.

We deal with two types of marked graph: those with tokens that can be only present or absent from an arrow, and those that, in addition, carry labels. For an unlabeled marked graph, a *firing* of a node is a relation between two markings: markings *M* and $M'$ are related by the firing of node *v* if the token number of $M'$ for each in-arrow of node *v* is 1 lower than the corresponding token number for *M*, and if the token number of $M'$ for each out-arrow of node *v* is 1 higher than the corresponding token number of *M*.

For a labeled marked graph some nodes are given rules that augment firings of nodes by specifying labels on tokens on out arrows as functions of labels on tokens of in-arrows.

A marking is called *live* if every node is either fireable, or can be made so through some sequence of firings.

A marking is called safe if no edge is assigned more than one token, and if no sequence of firings can bring two tokens or more to one edge (Commoner et al. 1971).

### Propositions for marked graphs

(Commoner et al. 1971, Theorem 12) states the following.

#### [Style1 Style2]Proposition 1

If a live marking M’ of a strong graph can produce M (through a sequence of firings), then M can produce M’.

Hence, “live markings of a strongly connected graph partition into equivalence classes. Let us refer to each equivalence class as a *family*” (Commoner et al. 1971). It follows that any marking of a family can be produced from any other marking by some sequence of firings.

We call the sum of token numbers over each arrow of a circuit the *token count of the circuit*. Because the firing of a node belonging to a circuit balances the decrease in the token number on the in-arrow of the node with the increase in the token number on the circuit out-arrow, we have the following

proposition.

#### [Style1 Style2]Proposition 2

The token count of a circuit is the same for markings related by firings, and hence the same for all markings in any family

(Commoner et al. 1971).

Note: the proofs in (Commoner et al. 1971) are asserted for graphs without loops, but they are valid also for graphs in our sense, i.e. simple graphs augmented by loops and multi-arrows.

### Propositions to do with changes in live and safe marked graphs into other live and safe marked graphs

We want to explore steps such as insertions of arrows and insertions or merging of nodes, done to a graph with a live and safe marking in such a way as to result in another graph with a live and safe marking. Our proofs assume basic knowledge of marked graphs with unlabeled tokens given in (Commoner et al. 1971).

#### [Style1 Style2]Proposition 3

Given any graph *G* with a live and safe marking *M*, let *a* be any arrow of *G*. Let the source of *a* be called *s* and the target of *a* be called *t*. Let $G'$ be the graph obtained by inserting a node, call it *v*, into *a*. That is, *a* is replaced by two arrows $a_1$ and $a_2$, one an in-arrow of a new node *v* and the other the out-arrow of *v*: $s \longrightarrow v \longrightarrow t$. Construct a marking $M'$ of $G'$ that agrees with *M* on arrows of *G*, places no token on $a_1$, and places a token on $a_2$ if and only if *M* assigns a token to *a*. Then $M'$ is live and safe.

#### Proof

The token count for $M'$ of every cycle of $G'$ is positive, and every arrow of $G'$ belongs to a circuit with token count 1. $\square$

#### [Style1 Style2]Proposition 4

Given two disjoint graphs $G_1$ and $G_2$ with live and safe markings $M_1$ and $M_2$, respectively, merging any node $v_1$ of $G_1$ with any node $v_2$ of $G_2$ results in a graph $G'$ with a live and safe marking $M'$ whose restriction to $G_j$ ($j\in \{1,2\}$) is $M_j$

#### Proof

Every cycle in $G'$ is contained in either $G_1$ or $G_2$ and so has positive token count; hence the marking $M'$ is live in $G'$. Every arrow in $G'$ belongs to either $G_1$ or $G_2$ and hence to a cycle with token count 1; hence $M'$ is safe. $\square$

#### [Style1 Style2]Proposition 5

Given a graph *G* with two distinct nodes *v* and *w* and a live and safe marking *M* for which both *v* and *w* are fireable, the graph $G'$ obtained by merging *v* and *w* has the marking *M* inherited from *G* as a live and safe marking in $G'$.

#### Proof

Merging of the nodes can create additional cycles, so the *M* is live in $G'$ if and only if the token count on these additional cycles is positive. It is positive because such a cycle goes through the merged node, and all the in-arrows of this node are marked, so that all cycles containing the merged node have positive token count. Now for safety. Because *M* is safe in *G*, any arrow *a* in $G'$ belongs to some cycle *C* of *G* with token count 1. *C* is also a cycle in $G'$. At most one of the nodes *v* and *w* can be in *C*. If neither is, then *C* has token count 1 in $G'$. If one of these nodes in *C*, then *C* still has token count 1 in $G'$. Hence the merge leaves *C* still a cycle in $G'$ with token count 1, so $G'$ is safe. $\square$

The above proposition raises the question relevant to the change portrayed in step 3 of fig. 4: Under what circumstances can a marking exist for which *v* and *w* are fireable. A sufficient but not necessary condition is given by the following.

#### [Style1 Style2]Proposition 6

Given two disjoint graphs $G_1$ and $G_2$, with live and safe families $\mathcal {M}_1$ and $\mathcal {M}_2$, respectively, and each containing at least two nodes, $v_j,w_j \in G_j$, $j \in \{1,2\}$. Let $G'$ be obtained by merging a node $v_1 \in G_1$ with the node $v_2 \in G_2$. Then there is a marking $M'$ of $G'$ that restricts to markings in $\mathcal {M}_1$ and $\mathcal {M}_2$ and that makes $w_1$ and $w_2$ both fireable in $G'$.

#### Proof

There are markings $M_j \in \mathcal {M_j}$ that make $w_j$ fireable, and $M_1\times M_2$ is a live and safe marking of $G'$. (One can ask if the merged node of $G'$ precludes any marking possible for the combination of $G_1$ and $G_2$; the answer is ‘no’ because there is a marking of $G'$ that makes the merged node fireable, and every other node of $G'$ belongs to $G_1$ or $G_2$, and by Theorem 7 of (Commoner et al. 1971) can be made fireable by some sequence of firings. $\square$

To address the splitting of a node needed to express the insertion of an amoeba into a slime-mold filament, we generalize the notion of a necklace used in (Murata and Koh 1980). In the immediate contest, let a link be any graph $G_j$, $j= 1, 2,\ldots , n$, such that $G_j$ has a live and safe marking and has two distinct nodes, say $v_j$ and $w_j$. Consider a chain of links obtained by merging link $w_1$ with $v_2$, $w_2$ with $v_3$, ..., $w_{n-1}$ with $v_n$. Then there is a marking of the chain of *n* links that makes both $w_n$ and $v_1$ fireable. For such a marking, merging $w_n$ with $v_1$ to close the chain results in a *necklace* of *n* links, and the necklace inherits a live and safe marking.

#### [Style1 Style2]Proposition 7

The graph obtained from a necklace of 2 or more links with a live and safe marking by splitting any *w*-*v* pair that joins links results in a chain that inherits a live and safe marking.

#### Proof

Every cycle of the chain is contained in the necklace and therefore has positive token count, so the marking of the chain is live. Every arrow of the chain belongs to a link as a subgraph with its own live and safe marking and hence to a cycle with token count 1, so the marking of the chain is safe. $\square$

#### [Style1 Style2]Proposition 8

Given a graph *G* with a live and safe marking that makes a node *v* fireable, inserting an arrow from *v* to any other node of *G* makes graph $G'$ that has a marking that is live. That marking is safe if and only if the inserted arrow is contained in a circuit in $G'$ with token count 1.

#### Proof

Liveness would fail for $G'$ only if $G'$ contained a circuit with null token count; but this is impossible because the only circuits in $G'$ not in *G* contain the inserted arrow, and every circuit that contains that arrow also contains an in-arrow to *v*, which is marked. The marking is safe if *a* is on a circuit with token count 1, because all the other arrows are on such a circuit in $G \subset G'$. $\square$

An example of an unsafe insertion is an arrow inserted diagonally into the graph Hex3-3 of (Myers and Madjid 2024) with a suitably chosen marking.

#### [Style1 Style2]Proposition 9

If a graph $G'$ with a live and safe marking *M* is obtainable by merging a node $v_1$ of a live and safe marked graph $G_1$ with a node $v_2$ of a disjoint live and safe marked graph $G_2$, then splitting the merged node retrieves $G_1$ and $G_2$, each with their live and safe markings.

#### Proof

For $j\in \{1,2\}$, every cycle in $G_j$ is contained in $G'$ and so $M_j$ has positive token count on all cycles of $G_j$, making $M_j$ live. As for safety, every arrow of $G_j$ is contained in a cycle of $G'$ with token count 1, and every cycle of $G'$ containing an arrow of $G_j$ is contained in $G_j$ $\square$

#### [Style1 Style2]Proposition 10

If a node *v* of a live and save marked graph *G* has a single in-arrow and a single out-arrow distinct from the in-arrow, and if *v* is not fireable, deletion of the in-arrow and merging of *v* with the source of the in-arrow of *v* produces a graph $G'$ with a live and safe marking.

#### Proof

Every cycle of $G'$ has positive token count and every arrow of $G'$ belongs to a cycle with token count 1. $\square$

#### [Style1 Style2]Proposition 11

If a necklace with a live and safe marking is obtainable by joining a pair of live and safe links as defined above, then the chain obtained by splitting a single node that joins a different pair of links has a live and safe marking, provided that all arrows incident to each node resulting from the split belong to a single link.

#### Proof

Any circuit in the chain is also in the live and safe necklace and thus has positive token count, so the marking of the chain is safe. Every arrow of the chain belongs to a link having a live and safe marking, and therefore belongs to a circuit with token count 1, and so the marking of the chain is safe. $\square$

## Contractions of fragments of live and safe marked graphs

In contrast to an unmarked graph, contracting a marked graph requires attention to the effect of the contraction on the markings. To preserve liveness and safety, we define contractions differently from the published morphisms of marked graphs and morphisms of elementary Petri nets found in (Winskel 1986; Wehler 2006; Bernardinello et al. 2013, 2014).

For a subgraph of a marked graph to be contractable, certain conditions on the marked graph and the subgraph must be satisfied. We first state the conditions for graphs with unlabeled tokens; then we make the extension to marked graphs with labeled tokens.

### Unlabeled tokens

Suppose $(G,\mathcal {M})$ is a marked graph with underlying graph *G* and a live and safe family $\mathcal {M}$. We present conditions under which a proper induced subgraph of *G* can be replaced by a single node. Let *H* be a proper induced subgraph of *G*, and suppose that the family $\mathcal {M}$:

- contains a marking $M_1$ having a token on each input arrow to *H* and having no token on any output arrow from *H* nor any tokens on arrows internal to *H*,
- contains another marking $M_2$ that has a token on each output arrow from *H* and has no token on any input arrow to *H* nor on arrows internal to *H*,
- makes the marking $M_2$ reachable from $M_1$ by firings only of nodes in *H*, and
- makes the marking $M_1$ reachable from $M_2$ without firing any node of *H*.

#### [Style1 Style2]Proposition 12

Under conditions (a), (b), (c), and (d) there is a mapping from $(G,\mathcal {M})$ to another unlabeled marked graph $(G',\mathcal {M_1'})$ having a live and safe marking, defined as follows. $G'$ is the graph obtained by shrinking *H* to a single node, call it *v*. $\mathcal {M_1'}$ contains $M_1'$ which has exactly the tokens of $M_1$.

#### Proof

By Theorem 3 of (Commoner et al. 1971), *G* is strong, so that $G'$ is also strong, and so can have a live and safe family. We need to prove that $M_1'$ belongs to such a family. For $M_1'$, *v* can fire, resulting in a marking $M'_2$, which matches $M_2$ on *G* and can thus reach $M_1'$. $\square$

### Contracting live and safe marked graphs with labeled tokens

Now we extend the procedure to deal with tokens labeled by arbitrary strings of bits. Under a mapping that contracts a subgraph *H* of *G* to a single node *w* of a smaller marked graph $G'$ having a live and safe marking, as above, the labeling rules for *w* *H* are as follows:

1. multiple arrows between any two nodes of $G'$ can be further contracted to a single arrow, but the bit strings carried by tokens on the arrows contracted are concatenated to form the bit string on the single arrow.
2. The in-arrow to $w\in H$ from a node $v\in V(G)\backslash V(H)$ is marked by a token carrying a concatenation of the strings of bits on the token on the in-arrows of *H* that come from *v*.
3. The labeling rule for the node *w* in $G'$ to which *H* contracts places strings of bits on tokens on an out-arrows of *w* that account for concatenation of input strings.

When *v* fires, the labels of tokens on out-arrows of *v* are logical functions of the labels of the tokens on its in-arrows. (If, in contrast, *H* were to contain a cycle, there would have to be a token on the cycle which acts a memory, and the labels of the tokens on out-arrows of *w* would depend not only on labels of the tokens on its in-arrows, but also on the labels of tokens previously placed on the in-arrows of *w*. I.e., the labeling rule would not be a Boolean function of the inputs alone.)
